# Left atrial function and fibrosis in lifelong endurance athletes: a cardiac magnetic resonance imaging study

**DOI:** 10.1007/s10554-025-03416-8

**Published:** 2025-05-21

**Authors:** Luuk H.G.A. Hopman, Jan-Peter Smedema, Jeroen Swart, Mayamiko J. Steenhoek, Irene M. Frenaij, Vijay Dahya, Marco J.W. Götte

**Affiliations:** 1 Cardiology Department, Amsterdam UMC, De Boelelaan 1118, Amsterdam, 1081 HV The Netherlands; 2 Netcare Blaauwberg Hospital, Cape Town, South Africa; 3 Health through Physical Activity, Lifestyle and Sport (HPALS) Research Centre, University of Cape Town, Cape Town, South Africa; 4 International Federation of Sports Medicine, Lausanne, Switzerland; 5 Cape Radiology, Constantiaberg Medi-Clinic, Cape Town, South Africa

**Keywords:** Atrial remodeling, Atrial fibrosis, Endurance athletes, Cardiac MRI

## Abstract

Participating in lifelong endurance sports is associated with an increased risk of atrial fibrillation (AF), potentially mediated through the process of left atrial (LA) remodeling which includes the progression of atrial fibrosis. This cross-sectional cohort study aimed to investigate LA remodeling including the presence and localization of LA fibrosis in lifelong endurance athletes. 42 master endurance athletes (76% male, age 54 ± 9 years), participating in Ironman races, (ultra) marathons and the Cape Epic Mountain Bike races, underwent clinical assessment with questionnaires, physical examination, and cardiac MRI (CMR). LA function, volumes, and left ventricular volumes were assessed. The extent of LA fibrosis, both global and regional, was derived from post-processed 3D late gadolinium-enhanced images using ADAS 3D. The median LA fibrotic burden as assessed by LGE-CMR was 2.5% (interquartile range, 1.1 to 7.6%). Fibrosis was heterogeneously distributed across the LA wall, with the highest burden observed along the left aspect of the posterior wall. There were no significant associations between LA fibrotic burden and atrial volume or functional parameters. Similarly, no associations were observed between fibrotic burden and sports level or training duration. LA fibrotic burden was significantly higher in participants who competed in long-distance mountain bike races versus participants who did not (7.3% [4.1–9.5] vs. 2.0% [0.5–5.6], *p* = 0.03). This study demonstrates the limited amount of LA fibrosis in lifelong master endurance athletes, with uneven distribution along the LA wall, most notably around the posterior side of the left inferior pulmonary vein. Participants engaged in long-distance mountain bike races exhibited significantly higher LA fibrotic burden, underscoring the potential impact of specific sports disciplines on LA remodeling, which may play a role in AF development.

**Supplementary Information:**

The online version contains supplementary material available at 10.1007/s10554-025-03416-8.

## Introduction

Extreme endurance sports have become increasingly popular in recent years, concomitantly with a growing number of enthusiasts who actively engage in activities such as marathon running, large cycling events, and triathlons [1]. While these sports offer numerous health related benefits, including improved cardiorespiratory health [2], they may also place participants at risk. Participating in high-intensity endurance sports can increase the risk of developing atrial fibrillation (AF) later in life, particularly in individuals who engage in these activities for extended periods of time [3]. Different studies show that the relationship between exercise dose and the relative risk of developing AF seems to be a U-shaped curve, as being inactive and being highly active are both risk factors [4, 5]. The prolonged and repetitive cardiac stress during endurance sports is hypothesized to lead to atrial remodeling, which can ultimately increase the risk of AF development [6]. 

Cardiac magnetic resonance (CMR) imaging is a powerful non-invasive imaging technique that can provide detailed information about the left atrial (LA) remodeling status [7]. This includes accurate measurements of atrial volumes, and assessment of atrial function using strain analysis [8]. Moreover, 3D high-resolution late gadolinium enhancement (LGE) CMR imaging can be used to investigate the extent and localization of fibrosis in the atrial wall [9]. Recent research has determined the relationship between the development of AF and the presence of atrial fibrosis, highlighting its importance in assessing AF risk [10, 11]. 

There is currently a scarcity of research on LA remodeling including fibrosis assessment in endurance athletes. To address this gap, we aimed to investigate LA remodeling using CMR in lifelong endurance athletes. Moreover, we assessed the presence and localization of LA fibrosis and we related LA fibrosis to sports activity, which may provide valuable insights into the pathophysiological mechanisms underlying AF risk in these athletes.

## Methods

This cross-sectional cohort study was conducted according to the principles outlined in the 1964 Declaration of Helsinki and its later amendments. Collection and management of data was approved by the local medical ethics committee. Clinical data were collected at Netcare Blaauwberg Hospital and CMR studies performed at Cape Radiology in Cape Town, South Africa between October 2021 and May 2023. Written informed consent was obtained from all individuals included in the study. The Amsterdam UMC served as a core lab for analysis of the CMR images.

### Study population

A group of 42 endurance athletes volunteered to participate in this study. All participants are highly trained athletes, with exceptional performances as completing multiple full Ironman races (4 km swimming, 180 km cycling and 42 km running), Two Oceans and Comrades ultra-marathons (respectively 56 km and 91 km), and Cape Epic Mountain Bike races (> 600 km, > 15000 m elevation). The inclusion criteria for study participation was habitual high-intensity exercise for at least 2 decades (> 4.5 h per week of exercise and > 3 sessions per week). Exclusion criteria were any known cardiac history or contra-indication to have a CMR scan. All participants underwent CMR imaging while still being active in their individual sport. Besides the contrast-enhanced CMR scan, all participants were clinically assessed with health questionnaires which included detailed athletic history.

### CMR protocol

All scans were performed using a 1.5 Tesla clinical MRI system (Siemens, Erlangen, Germany) with a 32-channel array coil. The CMR protocol included balanced steady state free precession cine imaging in long axis orientations (two-chamber and four-chamber view) and 3D high resolution LGE images. For cine imaging, typical in-plane resolution was 1.3 × 1.3 mm^2^ and acquisition parameters were as follows: repetition time, 41–47 ms; echo time, 1.6 ms; slice thickness, 5 mm; flip angle, 60–75°; matrix, 256 × 208 mm; temporal resolution, < 40 ms.

High resolution 3D LGE images were acquired using a navigator-based respiration- and ECG-gated inversion recovery prepared gradient echo pulse sequence applied approximately 13–17 min after administration of 0.15 mmol/kg gadoterate meglumine (Gd-BOPTA, MultiHance^®^) at a rate of 2.5 ml/s. The voxel size was 1.25 × 1.25 × 2.5 mm (reconstructed to 0.625 × 0.625 × 1.25 mm). Other typical sequence parameters were as follows: repetition time/ echo time was 5.2/2.4 ms; flip angle, 20°. Depending on the respiratory pattern and the heart rate of the participant, acquisition of the 3D LGE images took approximately 10–15 min.

### CMR data analysis

#### LA volume and global function

LA volume and global function assessment were conducted using Circle CVI^42^ (Version 5.11, Circle Cardiovascular Imaging, Inc, Calgary, Canada). Volumetric measurements for the LA and LV were obtained from two-chamber and four-chamber cine images using the biplanar method. LA volume (LAV) was further categorized into minimal (LAVmin) and maximal (LAVmax) volume. The total LA emptying fraction (LA EF) was calculated as ((LAVmax − LAVmin) × 100 / LAVmax). LAV index maximum (LAVImax) and LAV index minimum (LAVImin) were derived by dividing LAVmax and LAVmin by the body surface area.

#### LA strain assessment

Longitudinal LA strain analysis was performed using the Feature Tracking module in Circle CVI^42^ (Version 5.11, Circle Cardiovascular Imaging, Inc, Calgary, Canada). In the end-diastolic phase of the long-axis two-chamber and four-chamber cine images, endocardial and epicardial contours of the LA were traced. A combination of automated contour tracking with manual adjustments, if necessary, was utilized to obtain longitudinal strain measurements. These measurements were subdivided into LA reservoir strain, conduit strain, and contractile strain [12]. 

#### Quantification and analysis of LA fibrosis

Fibrosis assessment was performed using ADAS 3D LA (Adas3D Medical SL, Barcelona, Spain). Strict quality control measures were applied to the 3D LGE images by M.S. and L.H., and inadequate images were excluded from the analysis.

During post-processing, the LA wall including pulmonary vein (PV) extensions was manually segmented in multiple axial planes by drawing mid-atrial wall contours on the 3D LGE images. A 3D reconstruction of the LA was then automatically generated, with the LA appendage and PVs excluded based on their ostia defined as the point of deflection from the LA wall.

The LA was separated from the LV cavity using the mitral valve annulus, and enhancement at the mitral valve annulus was excluded for fibrosis analysis. Fibrosis presence and extent were determined using a default IIR threshold of 1.2 (1.2 times mean blood pool signal intensity) after normalizing signal intensity to the mean blood pool intensity according to the IIR-method [13]. 

**Fig. 1:**
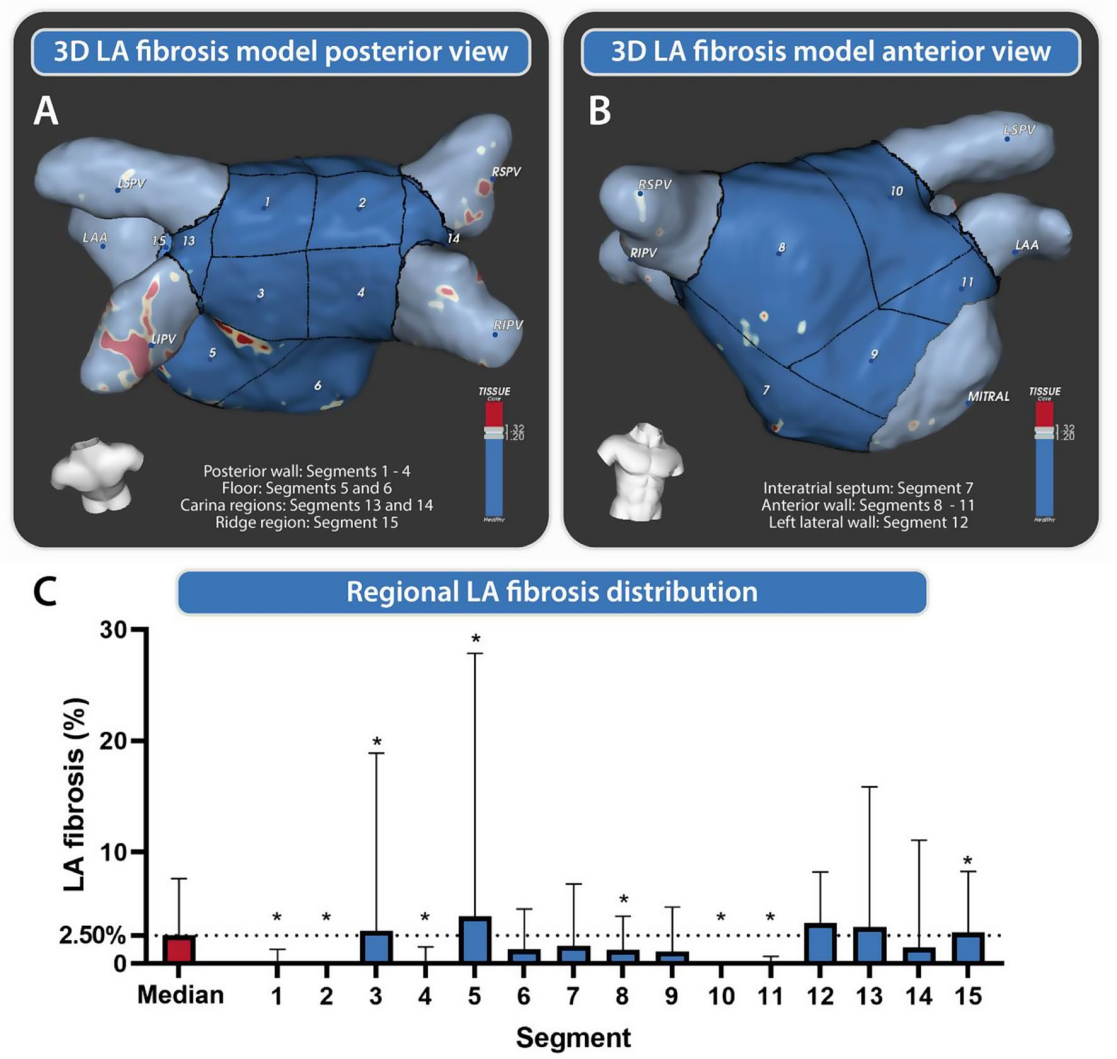
3D LA fibrosis model and regional LA fibrosis distribution. **A** and **B** show the segments 1 to 15 created through ADAS in posterior and anterior view. Red shows fibrosis patches, blue is healthy LA body and highlighted in light blue are the pulmonary veins, left atrial appendage and mitral valve. **C** is a boxplot describing the median fibrosis percentages for each segment. *Segment is significantly different than the median body fibrosis (*p* < 0.05)

Additionally, the LA was divided into 15 segments using an automated software process. The segments are defined in accordance with the method proposed by Benito et al. [14] Fig. 1 illustrates these segments, which were thoroughly reviewed and verified for accuracy. To assess the presence and extent of fibrosis in the LA, each segment underwent sequential analysis, allowing for precise calculations of fibrosis distribution throughout the LA.

### Statistical analysis

Results are presented as mean ± standard deviation (SD) or median (IQR) and categorical and ordinal variables are reported as frequencies or percentage. Normality of continuous data was assessed by inspection of histograms and Q-Q plots. To test for differences between two groups student’s t-test was used. Univariate linear regression models were created to find associations between continuous variables. When continuous data were not distributed normally the Mann-Whitney test was executed to test between two groups, the Kruskal-Wallis test was used to test between three groups, the Wilcoxon signed rank was used for multiple groups and Spearman’s correlation was computed to assess the relationship between variables. Statistical analysis was performed using SPSS Statistics v26 (IBM Corporation, Armonk, NY, USA). Data were considered significant with a p-value < 0.05.

## Results

The baseline characteristics of the study population are presented in Table 1. The mean age was 54 ± 9 years and 76% of the study population was male. The majority of participants were runners (62%), other participants were cyclists (17%) and triathletes (21%). The average number of exercise hours per week was 10.0 ± 5.4 h and the mean number of exercise years was 28.8 ± 11.4 years.

**Table 1:** Baseline characteristics of the study population (*n* = 42)

Demographics	
Age (years)	54 ± 9
Male gender Length (cm) Weight (kg)	32 (76%) 175.4 ± 7.1 71.9 ± 9.5
Body mass index (kg/m^2^) Body surface area (Mosteller)* (m^2^) Body fat (%)	23.3 ± 2.4 1.9 ± 0.2 12.8 ± 2.6
Discipline	
Runner Cyclist Triathlete Cape Epic mountain bike participants Comrades ultra-marathon participants Sports level	26 (62%) 7 (17%) 9 (21%) 9 (21%) 28 (67%)
Recreational Elite / Professional Exercise hours per week	33 (79%) 9 (21%) 10.0 ± 5.4
Exercise years Smoker Previous Smoker Hypertension Diabetes Alcohol use	28.8 ± 11.4 1 (2%) 8 (19%) 3 (7%) 0 (0%) 33 (79%)
On medication Statin Antidepressant Aspirin Beta-agonist bronchodilator Angiotensin-converting enzyme inhibitor Angiotensin II receptor blockers Amlodipine Family history of coronary artery disease	10 (24%) 5 (12%) 1 (2%) 2 (5%) 2 (5%) 1 (2%) 1 (2%) 1 (2%) 16 (38%)

All values are mean ± SD for continuous variables and number (%) for categorical variables. *Calculated by the Mosteller method ((height (cm) x weight (kg)/3600)^½^)

### Analysis of LA fibrosis and regional distribution

Good quality 3D LGE images for quantification of LA fibrosis were available in 37 participants (88%). The median (interquartile range) of global LA fibrotic burden was 2.5% (1.1–7.6). The regional assessment of fibrosis in these participants showed a non-uniform distribution of fibrosis across the LA wall (Fig. 1; Table 2). The segments 3, 5, and 15, corresponding to the posterior left inferior PV area, showed a significantly higher fibrotic burden than the median fibrotic burden (*p* = 0.02, *p* < 0.01 and *p* = 0.05, respectively), while segments 1, 2, 4, 8, 10, and 11 demonstrated significantly lower than the median fibrotic burden.

**Table 2:** Regional fibrosis quantification in lifelong endurance athletes (*n* = 37)

	Fibrosis % (median + IQR)	*p*-value
Total LA body Segment 1 Segment 2 Segment 3 Segment 4 Segment 5 Segment 6 Segment 7 Segment 8 Segment 9 Segment 10 Segment 11 Segment 12 Segment 13 Segment 14 Segment 15 Posterior wall Floor Anterior wall	2.5 [1.1–7.6] 0.0 [0.0–1.3] 0.0 [0.0–0.1] 2.9 [0.4–18.9] 0.0 [0.0–1.5] 4.2 [1.1–27.9] 1.3 [0.0–4.9] 1.6 [0.3–7.1] 1.2 [0.0–4.2] 1.1 [0.0–5.1] 0.0 [0.0–0.1] 0.0 [0.0–0.6] 3.6 [0.0–8.2] 3.3 [0.0–15.9] 1.5 [0–11.1] 2.8 [1.5–8.2] 1.4 [0.1–5.5] 2.9 [0.5–14.7] 1.1 [0.0–4.0]	**< 0.01** **< 0.01** **0.02** **< 0.01** **< 0.01** 0.06 0.48 **< 0.01** 0.05 **< 0.01** **< 0.01** 0.32 0.12 0.64 **0.05** 0.05 **0.01** **< 0.01**

Data are expressed as median [25th-75th percentile]. LA, Left atrium. Data in bold is considered significant (p-value < 0.05)

### LA fibrotic burden and clinical parameters

The median LA fibrotic burden was not significantly different between male and female participants (2.3% [0.9–6.6] vs. 4,1% [1.4–14.5], *p* = 0.28). Also, no relation was found between LA fibrosis and age (ρ=-0.19, *p* = 0.25), or Body Mass Index (ρ = 0.19, *p* = 0.25). Moreover, there was no significant relationship found between LA fibrotic burden and exercise hours (ρ = 0.17, *p* = 0.33) or exercise years (ρ=-0.22, *p* = 0.19).

**Fig. 2:**
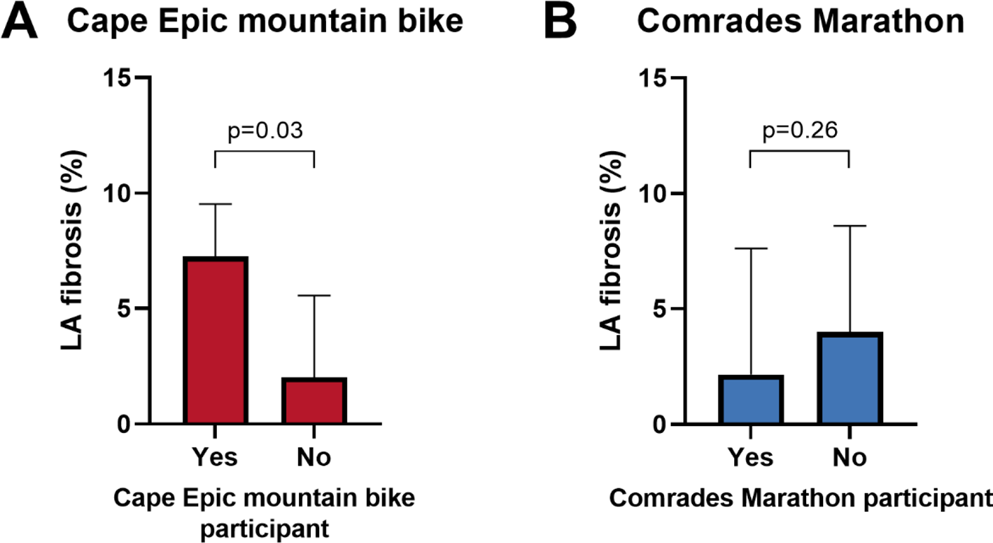
LA fibrosis score in Cape Epic mountain bike participants and Comrades marathon participants. (**A**) Boxplot showing LA fibrosis (%) of Cape Epic mountain bike participants compared to non-participants (**B**) Boxplot showing LA fibrosis (%) of Comrades marathon participants compared to non-participants

The median LA fibrotic burden between types of sport showed no significant difference between runners, cyclists and triathletes (runners: 2.2% [0.8–7.9], cyclists: 4.1% [0.9–7.7], triathletes: 3.2% [1.5–8.0], *p* = 0.95). However, participants who competed in long-distance mountain bike races (Cape Epic Races, > 600 km) demonstrated a higher LA fibrotic burden than the participants who did not (7.3% [4.1–9.5] vs. 2.0% [0.5–5.6], *p* = 0.03) (Fig. 2).

### LV and LA volume

Three participants were excluded from LV and LA volume analysis due to incomplete or low quality cine images (7%). Mean LV and LA volumetric and functional parameters are listed in Table 3. In summary, LV end diastolic volume (EDV) was 175.42 ± 26.85 mL, LV end systolic volume (ESV) was 64.82 ± 16.46 mL and the LV ejection fraction (LVEF) was 63.29 ± 5.55%. LAVImax was 46.14 ± 13.59 mL/m^2^ and the LA EF was 59.86 ± 7.09%. LA strain parameters were 16.76 ± 2.34% for reservoir strain, 9.01 ± 2.09% for conduit strain and 7.68 ± 1.72% for contractile strain.

**Table 3:** CMR-derived cardiac volume and function parameters (*n* = 39)

LV	
LV EDV (mL) LV ESV (mL) LV stroke volume (mL) LVEF (%)	175.42 ± 26.85 64.82 ± 16.46 107.90 ± 23.91 63.29 ± 5.55
LA	
LAV max (mL) LAV min (mL) LA stroke volume (mL) LA EF (%) LA reservoir strain (%) LA conduit strain (%) LA contractile strain (%) LAVImax (mL/m^2^) LAVImin (mL/m^2^) LAVImax (mL/m^2^) ≥ 50 mL/m^2^, n (%)	88.23 ± 20.11 35.93 ± 12.09 52.30 ± 11.24 59.86 ± 7.09 16.76 ± 2.34 9.01 ± 2.09 7.68 ± 1.72 46.14 ± 13.59 19.27 ± 6.62 15 (38%)

Values are expressed as mean ± SD. LV, left ventricle; EDV, End diastolic volume; ESV, end systolic value; LVEF, left ventricle ejection fraction; LA, left atrium; LAV max, left atrium maximum volume; LAV min, Left atrium minimal volume; LA EF, left atrial emptying fraction; LAVImax, left atrial volume index calculated with maximum left atrial volume; LAVImin, left atrial volume index calculated with minimal left atrial volume

**Fig. 3:**
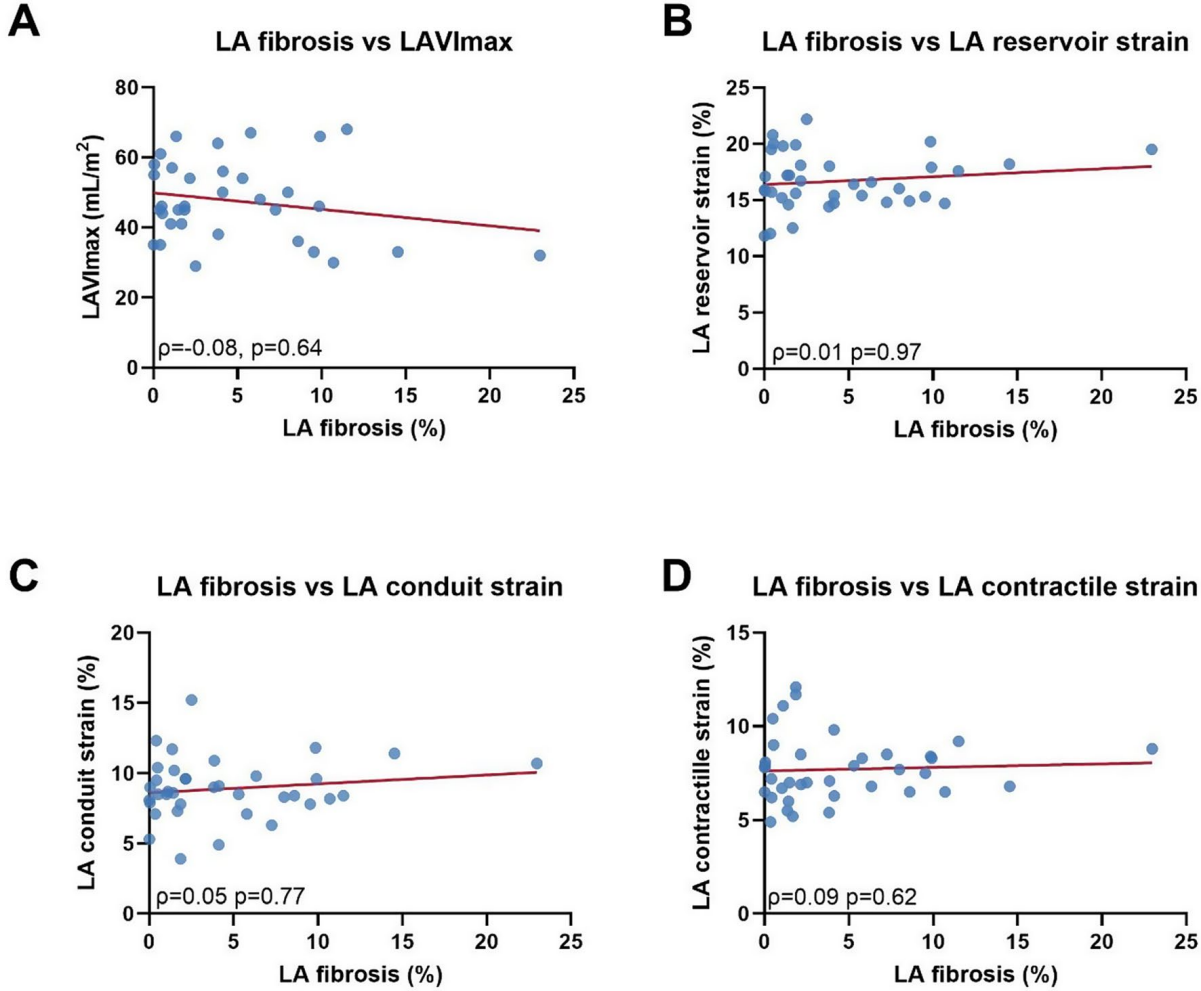
Correlation between LA fibrosis and LA function and volume. (**A**) LA fibrosis vs. LAVImax, (**B**) LA fibrosis vs. LA reservoir strain, (**C**) LA fibrosis vs. LA conduit strain and (**D**) LA fibrosis vs. LA contractile strain. Data are presented with correlation plots, spearman coefficient and p-value

There was no relationship found between atrial strain measurements and the LA fibrotic burden (reservoir strain; ρ = 0.01, *p* = 0.97, conduit strain; ρ = 0.05, *p* = 0.77 contractile strain; ρ = 0.09, *p* = 0.62) (Table 4; Fig. 3). Furthermore, no association was found between exercise hours per week and LA strain parameters (reservoir strain; *r* = 0.00, *p* > 0.99, conduit strain; *r*=-0.04, *p* = 0.83 contractile strain; *r* = 0.06, *p* = 0.72) or between exercise years and LA strain parameters (reservoir strain; *r* = 0.14, *p* = 0.41, conduit strain; *r*=-0.06, *p* = 0.72 contractile strain; *r* = 0.14, *p* = 0.41).

**Table 4:** The relation between LA fibrosis and clinical parameters

Independent variables	Correlation coefficient	*p*-value
Clinical parameters		
Age (years) Male gender (male / female) BMI (kg/m^2^) BSA (Mosteller)* (m^2^) Body fat (%) Exercise hours Exercise years Recreational level (recreational / elite)	-0.19 0.20 0.19 0.00 0.14 0.17 -0.22 -0.09	0.25 0.23 0.25 0.99 0.41 0.33 0.19 0.61
Cardiac parameters		
LVEF (%) LAVImax (mL/m^2^) LAVImin (mL/m^2^) Reservoir strain (%) Conduit strain (%) Contractile strain (%) LA EF (%)	-0.03 -0.08 -0.07 0.01 0.05 0.09 -0.05	0.87 0.64 0.69 0.97 0.77 0.62 0.79

Values are considered significant if p-value < 0.05. BMI, body mass index; BSA, body surface area; LVEF, left ventricular ejection fraction; LAVImax, left atrial volume index calculated with maximum left atrial volume; LAVImin, left atrial volume index calculated with minimal left atrial volume; LA EF, left atrial emptying fraction. *Calculated by the Mosteller method ((height (cm) x weight (kg)/3600)^½^)

## Discussion

This study demonstrated that the LA fibrotic burden as detected by LGE-CMR was low in a cohort of lifelong endurance athletes, while fibrosis exhibited a preferential regional distribution around the posterior side of the left inferior PV. Furthermore, no associations were found between LA fibrosis and LA volume or function parameters.

### Atrial fibrosis quantification in endurance athletes

Endurance sports have been linked to an increased risk of atrial arrhythmias, potentially driven by increased atrial filling pressures, LA dilation, and elevated inflammatory markers resulting in atrial remodeling [15]. Atrial remodeling, which involves the development of atrial fibrosis, is a recognized contributor to the risk of AF [10, 16]. CMR has been established as a precise and non-invasive imaging technique for effectively evaluating the remodeling status of the LA. Moreover, the past decade, literature has demonstrated the utility of LGE-CMR in visualizing and quantifying atrial fibrosis, mostly within cohorts of AF patients [11, 17]. Our study extended the application LGE-CMR to a unique cohort of lifelong endurance athletes, and found that the LA fibrotic burden was notably minimal (2.5%). Interestingly, according to the study by Benito et al., a similar LA fibrosis score was found in a cohort of healthy volunteers [18]. The limited presence of LA fibrosis in our athletic cohort implies that there is no discernible increase in fibrotic tissue compared to the general population. However, our findings are in discordance with a study by Peritz et al., in which LA fibrosis was also quantified in a group of endurance athletes. Their study reported an average LA fibrosis value of 9.6% in a control group, compared to a higher mean LA fibrosis burden of 15.5% in a cohort of 20 athletes mainly competing in cycling and Nordic skiing [19]. The significant variation in fibrosis values between studies might be ascribed to the disparate methodologies employed for LGE quantification. Peritz et al. utilized a quantification strategy that involves using healthy atrial tissue as a reference, incorporating a patient-specific dynamic threshold based on expert insights to identify fibrotic regions [19, 20]. In contrast, our investigation aligns with the methodology introduced by Benito et al. [18]., wherein a consistent thresholding technique is applied, employing a fixed cutoff value of 1.2 derived from the blood pool, as advocated by Khurram and colleagues [13]. This approach may provide a more standardized and reproducible means of quantifying atrial fibrosis [21]. Nevertheless, it is important to acknowledge the intrinsic limitations of atrial LGE imaging. Due to the thin atrial wall and limited spatial resolution, partial volume effects and difficulty in delineating the atrial wall remain challenges inherent to CMR-based fibrosis assessment. Moreover, the overall burden of LA fibrosis was low (2.5%) and a non-random regional distribution was observed. These findings may be interpreted in the context of physiological, rather than pathological, remodeling. Unlike studies such as DECAAF that focus on AF risk stratification, our analysis aimed to explore early, localized fibrotic adaptations associated with lifelong endurance training [11]. 

### Regional fibrosis distribution in endurance athletes: insights and potential mechanisms

Our research on the distribution of regional fibrosis in the LA has unveiled a distinct preference for localization, predominantly centered around the posterior left inferior PV area. Notably, our observations bear a remarkable resemblance to the findings of another study conducted by Benito et al. [14], highlighting identical segments exhibiting high fibrosis values among patients with AF. It is intriguing to note that while Benito’s study revealed a higher mean fibrosis burden, the distribution pattern of fibrosis is similar to that demonstrated in our athletes. This may imply that these athletes represent a subgroup at risk for developing AF. The presence of atrial fibrosis in the specific region of the posterior wall could potentially be linked to its close anatomical proximity to the descending aorta [22]. The impact of the aortic pulse, particularly intensified in athletes, could possibly result in lasting trauma and structural harm to the LA wall in this particular area [23]. This phenomenon could also explain the suggestion of increased levels of LA fibrosis observed in mountain bikers. Their distinctive riding position, and intensity of exertion with persistent high heart rates result in more prominent contact between the left lateral LA wall and the descending aorta. Although we identified a preferential distribution of fibrosis, we cannot draw firm conclusions regarding its functional significance due to the absence of rhythm monitoring data. Future studies incorporating ECG, Holter monitoring, or electrophysiology testing will be crucial to determine whether the location or extent of atrial fibrosis in endurance athletes predisposes to arrhythmias.

### Atrial remodeling and atrial function in endurance athletes

Scientific consensus exists regarding cardiac adaptations in individuals engaged in athletic activities [24]. By comparing our CMR derived atrial volume and function parameters with those derived from both healthy volunteers of similar age and patients diagnosed with AF, as elucidated in the study conducted by Hopman et al. [8], a noteworthy observation emerged. The volume and function measures obtained from the athletes in this study demonstrated an intermediary positioning between those found in healthy controls and AF patients (Supplementary Table 1). This distinctive positioning strongly implies the presence of atrial remodeling within the athletes, although less pronounced than in patients with AF.

In this study, no significant relation was found between atrial function parameters and LA fibrotic burden, which may reflect the overall low fibrosis burden in this athletic population. Previous studies have verified the relation between increased atrial fibrosis and impaired atrial function in AF patients [8, 25]. Interestingly, our findings propose a threshold effect wherein atrial function appears to be influenced only once a specific threshold of fibrosis is attained. This notion gains support from the fact that studies with AF patients exhibit notably elevated fibrosis levels compared to the endurance athletes enrolled in our analysis [18]. 

### Exercise hours and LA fibrotic burden in endurance athletes

We observed no significant correlation between the weekly exercise hours, life-time exercise hours and the burden of LA fibrosis. The absence of a significant correlation could potentially be attributed to the consistent and uniform level of exercise engagement among the participating athletes. Moreover, it is important to highlight that our study’s participants displayed a consistently high degree of physical activity upon entering the study assessments, reflecting their active lifestyle. This pronounced sporting lifestyle among our participants introduces an intriguing element to our analysis. It is worth noting that the presence of atrial LGE may not indisputably indicate fibrosis, but could alternatively be described as edema or inflammation in response to a prolonged, high-intensity endurance training [26, 27]. This raises the possibility that athletes who engage in exercise shortly before undergoing CMR imaging may exhibit heightened LGE values, which may not necessarily correlate with structural atrial remodeling. Given these considerations, the potential occurrence of edema and inflammation within the LA of athletes should be explored in future investigations. This research could offer valuable insights into discerning whether the observed LA LGE values genuinely correspond to fibrotic changes.

### Limitations and future studies

Several important limitations of this study must be acknowledged, particularly in light of the technical and design-related concerns raised. Primarily, the modest size of our study cohort introduces potential constraints in investigating certain correlations and distinctions between groups. Subgroup analyses, including the comparison of mountain bikers and non-mountain bikers, were exploratory in nature and should be interpreted with caution. We emphasize that this study is hypothesis-generating. Furthermore, the lack of a non-athletic control group limits our ability to make definitive conclusions about whether the observed fibrotic burden exceeds that seen in the general population. While prior studies using comparable LGE-CMR methods in healthy controls have reported similarly low levels of fibrosis, direct comparison would have added value [18]. Therefore, future research should use a larger cohort with a control group to demonstrate if the LA fibrosis burden is more evident in athletes than in controls.

Secondly, our participants exhibited inherent heterogeneity in sporting activities. The potential overlap between sports, such as cyclists engaging in running or vice versa, hinders accurate inter-sport comparisons. Future research endeavors should proactively delineate distinct sports categories in advance, selecting disciplines with minimal overlap, to facilitate more meaningful contrasts.

Thirdly, the accuracy of exercise hours and duration, as provided in the questionnaires, could be subject to inaccuracies. The integration of heart rate sensors emerges as a promising alternative. Given their prevalent use among athletes and capacity to provide insights into training intensity and duration, heart rate sensors offer a potential avenue for more precise data collection.

Finally, a potential source of bias stems from the exclusion of athletes with prior cardiac history. This selective approach might have contributed to an underestimation of fibrosis values and superior atrial function parameters compared to what might be anticipated for athletes of this age and endurance level.

## Conclusions

This study highlights a low fibrotic burden in master endurance athletes. However, athletes exhibit a comparable distribution of fibrosis as observed in AF patients, indicating that the atrial remodeling seen in endurance athletes might represent an early stage of the remodeling process observed in AF patients. Nevertheless, the study did not establish a significant correlation between atrial fibrosis and atrial function parameters, suggesting that the extent of fibrosis in endurance athletes has not yet reached a threshold that adversely affects LA function. Future studies combining CMR imaging with rhythm monitoring and including non-athletic control groups will be essential to clarify whether the increased AF risk in endurance athletes is related to the extent or progression of atrial fibrosis, and whether specific patterns or locations of fibrosis predict arrhythmogenic remodeling.

## Electronic supplementary material

Below is the link to the electronic supplementary material.

Supplementary Material 1

## Data Availability

The data underlying this article will be shared on reasonable request to the corresponding author.

## Abbreviations

AF: Atrial fibrillation
CMR: Cardiac magnetic resonance
LA: Left atrium
LAV: Left atrial volume
LAVi: Left atrial volume index
LAVimax: Left atrial volume index max
LAVimin: Left atrial volume index min
LA EF: Left atrial emptying fraction
LGE: Late gadolinium enhancement
LV: Left ventricle
PV: Pulmonary vein
