# Supplementary material for: Left atrial function and fibrosis in lifelong endurance athletes: a cardiac magnetic resonance imaging study
Source: Int J Cardiovasc Imaging. 2025 May 21;41(7):1321–30. doi: 10.1007/s10554-025-03416-8 (PMC12241282; doi:10.1007/s10554-025-03416-8)
Supplement: Supplementary file 1 — Supplementary Material 1 [file 10554_2025_3416_MOESM1_ESM.docx]

**SUPPLEMENTARY MATERIAL**

| **Table S1:** CMR-derived cardiac volume and function parameters compared with study  conducted by Hopman et al. (1). | | | |
| --- | --- | --- | --- |
|  | **Endurance athletes (n=39)**  **(Current study)** | AF patients (n= 94)  (Hopman et al.) | Healthy controls (n= 19)  (Hopman et al.) |
| Age  LV  LV EDV (mL)  LV ESV (mL)  LVEF (%)  LA  LAV max (mL)  LAV min (mL)  LA EF (%)  LA reservoir strain (%)  LA conduit strain (%)  LA contractile strain (%)  LAVImax (mL/m^2^) | **54 ± 9**  **175 ± 27**  **65 ± 16**  **63 ± 6**  **88 ± 20**  **36 ± 12**  **60 ± 7**  **17 ± 2.3**  **9.0 ± 2.1**  **7.7 ± 1.7**  **46 ± 14** | 60 ± 9  168 ± 42  69 ± 22  59 ± 7  100 ± 32  50 ± 28  52 ± 13  15.9 ± 3.8  8.7 ± 2.7  7.2 ± 2.3  49 ± 15 | 58 ± 4  146 ± 27  55 ± 12  62 ± 5  70 ± 15  26 ± 11  64 ± 8  21.1 ± 3.6  12.6 ± 2.5  8.6 ± 2.2  37 ± 8 |
| Values are expressed as mean ± SD. LV, left ventricle; EDV, End diastolic volume; ESV, end systolic value; LVEF, left ventricle ejection fraction; LA, left atrium; LAV max, left atrium maximum volume; LAV min, Left atrium minimal volume; LA EF, left atrial emptying fraction; LAVImax, left atrial volume index calculated with maximum left atrial volume | | | |

1. Hopman L, Mulder MJ, van der Laan AM, Demirkiran A, Bhagirath P, van Rossum AC, et al. Impaired left atrial reservoir and conduit strain in patients with atrial fibrillation and extensive left atrial fibrosis. J Cardiovasc Magn Reson. 2021;23(1):131.
